# Is orbital floor a reliable and useful surgical landmark in endoscopic endonasal surgery?: a systematic review

**DOI:** 10.1186/s12901-018-0060-5

**Published:** 2018-07-24

**Authors:** Baharudin Abdullah, Chew Shiun Chuen, Salina Husain, Kornkiat Snidvongs, De Yun Wang

**Affiliations:** 1 Department of Otorhinolaryngology-Head & Neck Surgery, School of Medical Sciences, Universiti Sains Malaysia Health Campus, 16150, Kubang Kerian, Kota Bharu, Kelantan Malaysia; 2 Department of Otorhinolaryngology- Head & Neck Surgery, Universiti Kebangsaan Malaysia Medical Centre, Jalan Yaacob Latif, Bandar Tun Razak, Kuala Lumpur, Malaysia; 3 Department of Otolaryngology Head and Neck Surgery, Faculty of Medicine, Chulalongkorn University and King Chulalongkorn Memorial Hospital, Bangkok, Thailand; 4 Department of Otolaryngology, Yong Loo Lin School of Medicine, National University of Singapore, Singapore, 119228 Singapore

**Keywords:** Orbital floor, Skull base, Sphenoid, Ethmoid, Endoscopic sinus surgery

## Abstract

**Background:**

The orbital floor is considered as an important intraoperative reference point in endoscopic sinonasal surgery. The aim of this review is to evaluate its reliability and usefulness as a surgical landmark in endoscopic endonasal surgery.

**Methods:**

A literature search was performed on electronic databases, namely PUBMED. The following keywords were used either individually or in combination: orbital floor; maxillary sinus roof; endoscopic skull base surgery; endoscopic sinus surgery. Studies that used orbital floor as a landmark for endoscopic endonasal surgery were included in the analysis. In addition, relevant articles were identified from the references of articles that had been retrieved. The search was conducted over a period of 6 months between 1st June 2017 and 16th December 2017.

**Results:**

One thousand seven hundred forty-three articles were retrieved from the electronic databases. Only 5 articles that met the review criteria were selected. Five studies of the orbital floor (or the maxillary sinus roof) were reviewed, one was a cadaveric study while another 4 were computed tomographic study of the paranasal sinuses. All studies were of level III evidence and consists of a total number of 948 nostrils. All studies showed the orbital floor was below the anterior skull base irrespective of the populations. The orbital floor serves as a guide for safe entry into posterior ethmoids and sphenoid sinus.

**Conclusions:**

The orbital floor is a reliable and useful surgical landmark in endoscopic endonasal surgery. In revision cases or advanced disease, the normal landmarks can be distorted or absent and the orbital floor serves as a reference point for surgeons to avoid any unintentional injury to the skull base, the internal carotid artery and other critical structures.

## Background

Endoscopic approach to address chronic sinus disease is deemed a minimally invasive surgical technique. The goals of endoscopic sinus surgery (ESS) is to address the affected sinuses to restore and improve the drainage system [1]. It was achieved by opening the natural ostium of the sinus and preserving the mucosa for the natural process of mucociliary clearance to take place. Secondly, the goal of opening the cavities is to facilitate clinical procedure such as debridement or taking cultures when required. The third goal is the removal of all diseased cells to gain access to the last layer of mucosa. The fourth goal aims to create the access for long term topical management, including diseased cells and non-diseased cells [2, 3]. Thus, a complete sphenoethmoidectomy may allow access to the mucosa along the skull base and orbit and facilitates irrigation, topical medication and clinical surveillance.

Most importantly, the main goal of treatment is primarily to improve patients’ quality of life, and causing an avoidable complication is unwarranted [1]. When performing an ESS in both primary and revision surgeries, the usual anatomical landmarks are crucial as reference points. However, in revision cases or advanced disease the normal landmarks that surgeon used as a guide can be distorted or absent. Thus, dissection becomes difficult and potentially hazardous, especially when the surgeons do not have sufficient understanding of the anatomy to navigate their way safely through the sinonasal cavity. As a result, surgery might be incomplete or inadequate and the potential risk of complication is higher [1].

Above all else, to operate confidently and safely while performing an endoscopic surgery, surgeons must be able to navigate in the restricted 3-dimensional area by viewing a 2-dimensional screen with the guide of the constant landmarks [1]. A well trained surgeons should be comfortable with using multiple landmarks as their guide. Relying on just one landmark may not be possible, for example the middle turbinate could have been removed previously in the cases of revision surgery. However, there are the landmarks that are almost always present and identifiable regardless of the number of previous surgeries or extent of nasal polyposis such as the nasal floor, the arch of posterior choanae, the septum (or remnant of the septum), nasolacrimal convexity, medial orbital floor, posterior maxillary wall, medial orbital wall and the fovea ethmoidalis [1, 4]. In the context of addressing the skull base, some of these structures are deemed to be too superior or inferior as reference during surgery. Besides, some structures required a multiplanar computed tomography of paranasal sinuses (CT PNS) for identification, unlike the orbital floor (OF) which is readily detectable. May et al. [5] had described the maxillary ostium as an important landmark which is constantly below the OF which highlight the use of OF as a reference point for endoscopic sinus surgery.

**Fig. 1:**
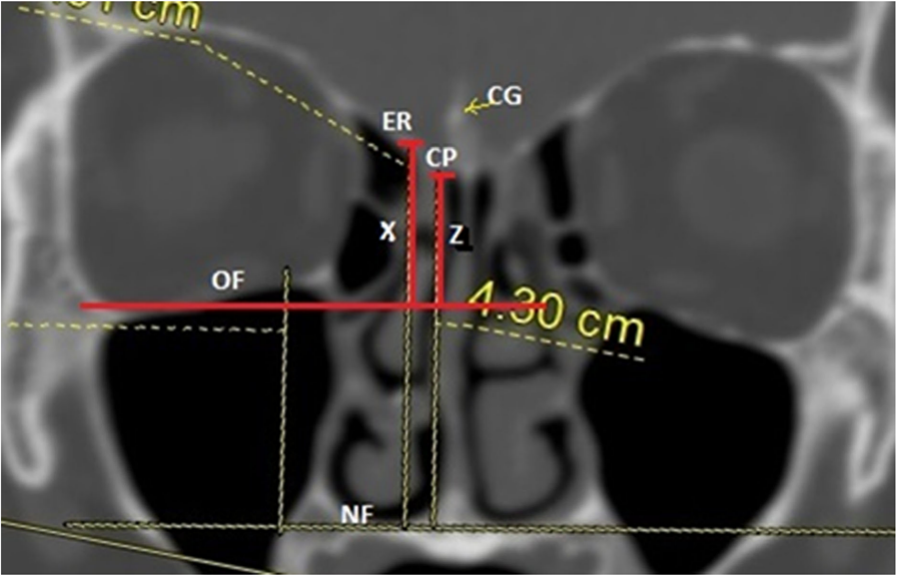
CT scan of paranasal sinus (coronal view) showing the relation of orbital floor from the other anatomical landmarks. (CG- crista galli; ER- ethmoid roof; CP- cribriform plate; OF- orbital floor; NF- nasal floor; X-ethmoid roof to orbital floor; Z- cribriform plate to orbital floor)

The complications of endoscopic surgery categorised according to severity as minor or major; and according to the time of appearance as immediate or delayed [6]. Minor complications ranged from 2 to 21% of cases such as synechiae, crusts, minor bleeding, alteration of dental sensitivity, edema, periorbital ecchymosis, stenosis of sinus ostia, hyposmia and epiphora [7, 8]. The major catastrophic complications are vascular injury, orbital and intracranial complications which vary from 1 to 3% [9]. The most common immediate complications are CSF leak, intraoperative bleeding, orbital hematoma and injury to the brain [10]. Progressive loss of vision or smell, meningitis, bleeding, synechiae and infection are the delayed complications [6]. These risks are exacerbated in revision surgery in which the usual anatomic landmarks may be distorted or absent [11]. A fine-cut (0.5 to 1 mm) CT PNS is essential to provide information regarding patient’s anatomical variants and the degree of distortions by the disease process to ensure a safe surgery (Fig. 1).

**Fig. 2:**
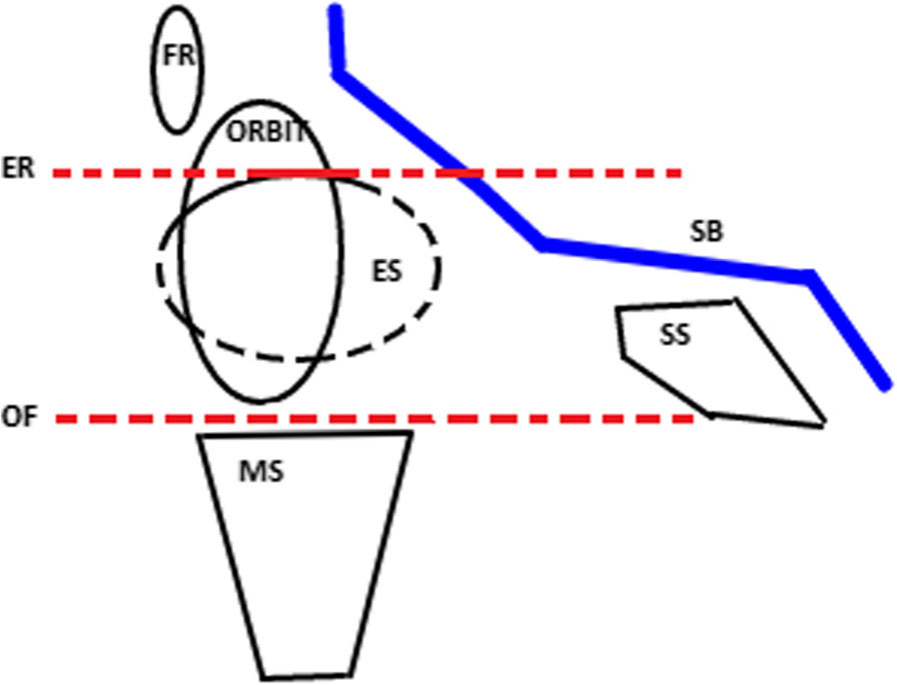
Sagittal view of the orbital floor in relations with the skull base. (FR-frontal sinus, ORBIT-orbit, ES- ethmoid sinus, MS - maxilla sinus, SS- sphenoid sinus, ER- level of the ethmoid roof, OF- level of the orbital floor and SB- skull base (blue))

The OF is considered as an important intraoperative reference point as a guide during endoscopic sinonasal surgery [12, 14]. The OF comprises mostly of the orbital plate of the maxilla and the tiny orbital plate of the palatine bone posteriorly and by the inferior orbital process of the zygoma anterolaterally. It is shorter in its anteroposterior extent than the three other orbital walls and terminates in the inferior orbital fissure in front of the orbital apex. It is thin-walled and forms the superior boundary of the maxillary sinus. Harvey et al. [13], Wuttiwongsanon et al. [14] and Lee et al. [15] demonstrated that the OF is persistently below the skull base and it can be used as a fixed anatomical landmark. While other landmarks that have been traditionally described like the middle or superior turbinates could be distorted by pathology or removed during surgery, the OF is seldom affected. There can be significant variability in the degree of incline of the skull base, which may narrow the vertical dissection distance during posterior ethmoidectomy or transethmoid sphenoidotomy (Fig. 2). Therefore, by staying below or at the level of the OF as the dissection proceeds posteriorly, injury to the skull base is avoided [13, 16]. The aim of this systematic review is to determine the usefulness and reliability of OF as a fixed anatomical landmark in endoscopic endonasal surgery.

## Methods

A search was performed on electronic databases, namely PUBMED. The following keywords were used either individually or in combination: orbital floor; maxillary sinus roof; endoscopic skull base surgery; endoscopic sinus surgery. Studies that used OF as a landmark for endoscopic endonasal surgery were included in the analysis. Some relevant articles related to this review were identified by reviewing the references of articles that had been retrieved. Certain information provided in the articles were counter checked and compared with standard textbooks. The search was conducted intermittently over a period of 6 months between 1st June 2017 and 16th December 2017 in accordance with the Preferred Reporting Items for Systematic Reviews and Meta-Analyses (PRISMA) guidelines [17] and the Cochrane Handbook [18] when appropriate.

## Results

### Selections of studies

**Fig. 3:**
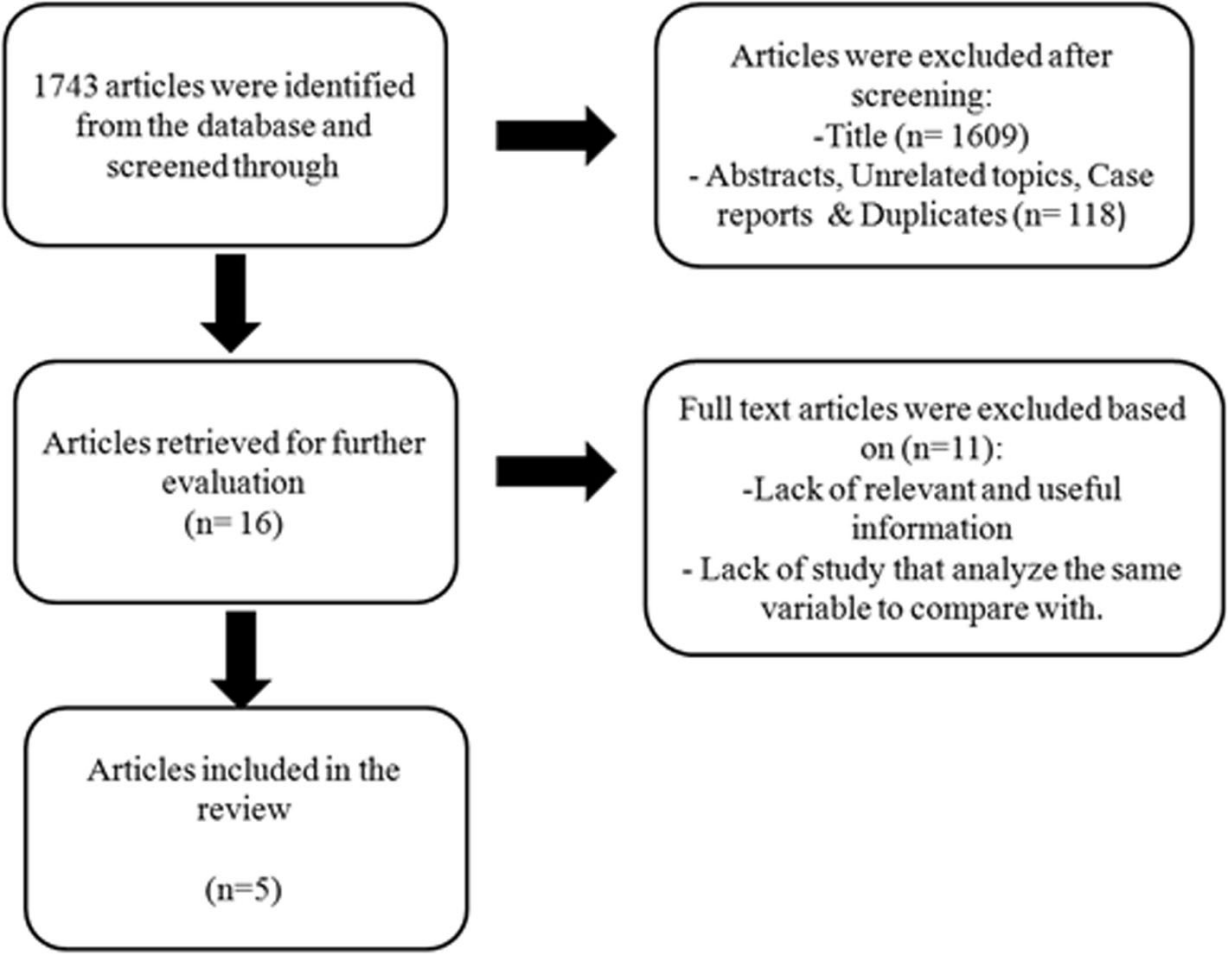
Flow diagram showed how the relevant articles to this review were selected

One thousand seven hundred forty-three articles were retrieved from the electronic databases. One thousand six hundred nine articles were excluded after screening the title as they did not meet the review criteria**.** After screening the abstracts, 118 articles which were either case reports or unrelated topics were excluded. Out of the 16 full text articles retrieved, 5 articles were selected and the remainders were excluded from this review owing to lack of relevant information or lack of similar variables for comparison (Fig. 3). All studies were of level III evidence and consists of a total number of 948 nostrils (Table 1).

**Table 1:** Overall summary of studies included in the review

Study	Year of study	Type of study	No of Sides
Casiano et al. [12]	2001	Cadaver	18
Harvey et al. [13]	2010	CT PNS^a^	300
Lee et al. [15]	2012	CT PNS^a^	100
Wuttiwongsanon et al. [14]	2015	CT PNS^a^	300
Lee [19]	2017	CT PNS^a^	230
		Total	948

^a^*CT PNS* computed tomography of the paranasal sinuses

### Radiological studies

Harvey et al. [13], Wuttiwongsanon et al. [14], Lee et al. [15] and Lee [19] conducted studies on Australian and American, Thai, Canadian and Korean populations respectively (Table 2). All studies were based on CT PNS that documented the OF height relative to the nasal floor. The reference point for the measurements was at the medial extent of the orbital floor. The average OF height from the nasal floor documented by Harvey et al. [13], Wuttiwongsanon et al. [14], Lee et al. [15] & Lee [19] was 33.9 ± 3.0, 35.2 ± 3.4, 33.45 ± 2.83 and 33.83 ± 3.4 respectively. All the measurements were relatively constant despite being done in different populations (Table 3).

**Table 2:** Ethnicity and type of study conducted

Study	Year	Type	Region	Ethnic	No. of Subject		
					Total	Right	Left
Casiano et al. [12]^a^	2001	Cadaver	USA	Caucasian	18	–	–
Harvey et al. [13]	2010	CT PNS^b^	USA & Australia	Caucasian	300	150	150
Lee et al. [15]	2012	CT PNS^b^	Canada	Caucasian	100	50	50
Wuttiwongsanon et al. [14]	2015	CT PNS^b^	Thailand	Asian	300	150	150
Lee [19]	2017	CT PNS^b^	Korea	Asian	230	115	115
Total					948		

^a^right and left sides were not specified

^b^CT PNS- computed tomography of the paranasal sinuses

**Table 3:** Vertical height of the sphenoid ostium from the orbital floor

Landmarks	Lee et al. [13] Mean (mm) ± SD	Lee [16] Mean (mm) ± SD
Height of maxillary sinus roof	33.45 ± 2.83	33.83 ± 3.40
Maxillary sinus roof to posterior ethmoid roof	14.08 ± 3.03	Not measured
Maxillary sinus roof to sphenoid ostium	2.76 ± 2.80	1.79 ± 3.09
Maxillary sinus roof to sphenoid roof	12.18 ± 3.20	12.02 ± 2.93
Maxillary sinus roof to sphenoid floor	5.94 ± 2.94	6.18 ± 2.88

### Cadeveric study

Casiano et al. [12] measured 18 sides of cadaveric heads by performing endoscopic measurements of OF distance to the skull base and other vital anatomical structures such as the anterior ethmoidal artery (AEA), carotid artery and optic nerve (Table 2). Remarkably, all these structures were at a significant distance from the OF. Their results showed the mean distance of OF to carotid artery was 16.94 ± 4.15 mm, OF to optic nerve was 16.03 ± 2.56 mm, OF to mid-ethmoid was 16.44 ± 2.51 mm and OF to AEA was 21.56 ± 2.66 mm.

### Height of OF related to height of anterior skull base

Harvey et al. [13] and Wuttiwongsanon et al. [14] measured CT PNS of 150 subjects with 300 sides. The mean vertical height of the landmarks from the nasal floor to cribriform, ethmoid roof and sphenoid roof were 44.0 ± 3.7, 48.4 ± 4.5, 44.9 ± 3.7 respectively in Caucasian [13] population and 46.4 ± 3.6, 49.3 ± 3.8 and 45.7 ± 3.7 respectively in Asian [14] population. In addition, the mean vertical heights of the OF to the anterior skull base specifically cribriform, ethmoid roof and sphenoid roof were measured. They were 10.1 ± 2.7, 14.5 ± 3.5 and 11.0 ± 2.9 respectively in Caucasian [13] population and 11.2 ± 2.5, 14.1 ± 3.1 and 10.5 ± 3.3 respectively in Asian [14] population.

### Height of the OF related to sphenoid

Lee et al. [15] measured CT PNS from 50 subjects with a total of 100 sides, whereas Lee [19] measured 230 sides from 115 subjects. The two studies showed relative to the OF, the mean height of the sphenoid roof was 12.18 ± 3.20 mm and 12.02 ± 2.93 mm respectively and the mean height of the sphenoid floor was 5.94 ± 2.94 mm and 6.18 ± 2.88 mm respectively. In addition, the mean height of the natural sphenoid ostium in relation to the OF was measured and documented as 2.76 ± 2.80 mm and 1.79 ± 3.09 respectively (Table 3).

### The OF distance to vital structures: AEA, carotid artery and optic nerve

Direct and endoscopic measurements by Casiano et al. [12] had established that AEA, carotid artery and the optic nerve were at a significant distance from OF. Their results showed the mean distance of OF to AEA, carotid artery and the optic nerve were 21.89 ± 2.42 15.89 ± 3.96 and 15.22 ± 2.42 respectively from direct measurement and 21.56 ± 2.66, 16.94 ± 4.15 and 16.03 ± 2.56 respectively from endoscopic measurement.

### Difference (or similarity) across population

In Asians, the distance of OF to the cribriform was longer and the distance of OF to the sphenoid roof was shorter as compared to Caucasians [14]. All studies showed that OF was below the anterior skull base irrespective of the populations [12–15, 19] (Table 4). Overall, there was no significant height difference between the left and right skull base documented [14, 15, 19].

**Table 4:** Mean orbital floor height to the key surgical landmarks

Landmarks	Harvey et al. [13] Mean(mm) ± SD	Wuttiwongsanon et al. [14] Mean(mm) ± SD	Lee et al. [15] Mean(mm) ± SD	Lee [19] Mean(mm) ± SD
Orbital floor to nasal floor	33.9 ± 3.0	35.2 ± 3.4	33.45 ± 2.83	33.83 ± 3.40
Orbital floor to sphenoid roof	11.0 ± 2.9	10.5 ± 3.3	12.18 ± 3.20	12.02 ± 2.93
Orbital floor to ethmoid roof	14.5 ± 3.5	14.1 ± 3.1	14.08 ± 3.03	Not measured

## Discussion

The introduction of intraoperative navigation in the form of image-guided surgery (IGS) is often beneficial in revision ESS, and there are multiple systems available commercially [20]. However, IGS is a tool that may be inaccurate at times and may fail during the course of an operation and a properly trained personnel usually are required. Thus, surgeons still need to be guided by familiar landmarks during ESS. The study by Casiano et al. [12] had showed OF was at a significant distance from carotid artery, optic nerve, ethmoid and AEA. The reference points provide even the most inexperienced surgeon with precise anatomic localization within the paranasal sinuses. Thus, OF serves as a useful landmark to avoid any unintentional injury to those structures. In addition, the OF serves as an important landmark to provide a safe route of entry into the sphenoid when all other anatomic features have been distorted as once the sphenoid roof is located, the remainder of the skull base can be identified by working from posterior to anterior [16]. Furthermore, the minimum measurement of the mean vertical heights of more than 10 mm from OF to those structures [12], allows the use of many common surgical instruments in this restricted area.

The study conducted by Harvey et al. [13] involved two different populations; Australians and Americans. By using multiplanar CT PNS, the vertical height from nasal floor and OF to the surgical landmarks: the cribriform, ethmoid and sphenoid roofs were measured. They showed that OF was 100% below the pre-mentioned surgical landmarks in both groups. Interestingly, they also found even in patients with a very high and well-pneumatised maxillary sinus when the distances to the critical anatomy were reduced, the OF was still constantly below the skull base.

Wuttiwongsanon et al. [14] compared the skull base height between two populations; Caucasians and Asians. A relative difference in the skull base heights between these populations were documented. The Asian population had a longer distance from OF to cribriform and a shorter distance from OF to sphenoid roof as compared to Caucasians. However, the rule of staying below the orbital floor to prevent skull base injury is still applicable to the different ethnic groups [13, 14]. The maxillary sinus is the most pneumatized paranasal sinus and it’s the largest in size. They resemble a quadrilateral pyramidal cavity extending into the bodies of the maxilla [21, 22]. Lee et al. [23] showed that there was a significant difference along gender and ethnic lines in maxillary sinus volume but there was very little difference in the shape of the maxillary sinus. The sinus appears to preserve a parabolic-hyperbolic shape despite quite marked variations in volume. Thus, despite the ethnic anatomical difference, OF is rather fixed in its location as the shape of the maxillary sinus is constant.

A similar study based on CT PNS was conducted by Lee et al. [15]. Their study showed the natural sphenoid ostium was located at a vertical height of 2.76 mm superior to OF. By acknowledging this relation, OF may be used as a guide for locating the height of the natural sphenoid opening while performing transethmoid sphenoidotomy. In the presence of excessive bleeding or abundant inflamed mucosa obscuring the view of the natural sphenoid ostium, this will become extremely useful. Lee et al. [15] also looked at variations of the maxillary sinus size and changes in the posterior ethmoid vertical height by studying the ratio of the maxillary sinus to the posterior ethmoid height. Their study showed the mean ratio was 2.49, with the distribution of calculated ratios reveals a distribution with a wide range from 1.36 to 4.34. The lower ratios determined a wider posterior ethmoid dissection distance and greater room for safety away from the skull base, whereas high ratios indicates a narrow posterior ethmoid dissection distance. This led them to propose a classification scheme utilizing the ratio of the maxillary sinus to the posterior ethmoid height: Class I is less than 2, Class II is from 2 to 3, and Class III is more than 3. Approximately, 64% of all individuals are in class II and 18% of individuals in classes I and III [15]. Further studies may be required to determine the usefulness of this classification scheme in the evaluation of preoperative CT PNS.

Lee [19] in another study demonstrated similar comparable findings. In addition, the study showed that the posterior maxillary sinus wall can be used as a fixed landmark as well. The study measured the distance between the sphenoid ostium relative to the posterior maxillary sinus wall, with the mean distance of 0.78 mm obtained. The study found 44.4% of the sphenoid ostium was located posterior from the coronal plane of the posterior wall of the maxillary sinus, followed by 29.3% at the same level and 26.3% anterior to the posterior wall of the maxillary sinus [19]. One distinct and homogenous finding by all these investigators revealed that there was no significant height differences between left and right skull base [14, 15, 19]. Other studies had shown the posterior ethmoidal roof is relatively constant between both sides [24] whereas asymmetry is more commonly noted in the anterior ethmoid roof [25].

**Fig. 4:**
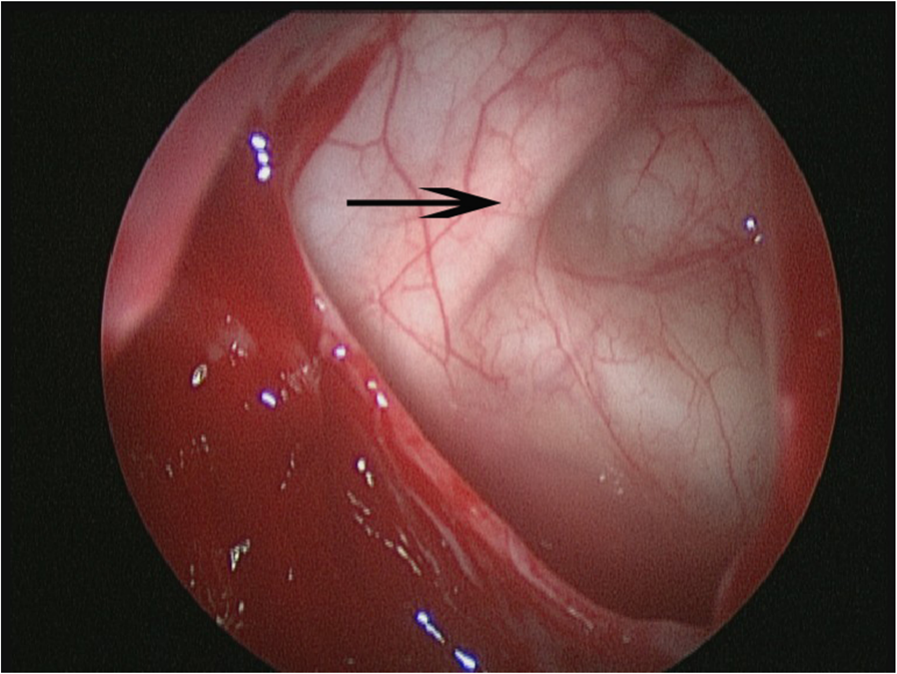
Endoscopic view of infraorbital nerve course at the orbital floor after maxillary anstrostomy

Another important use of OF is in the identification of the infraorbital nerve (ION). The ION located in OF is a useful superficial landmark for identifying deeper structures such as the trigeminal nerve (V2), the pterygopalatine fossa and the cavernous sinus [26]. It has been observed that upon entrance to the maxillary sinus, ION (orbitomaxillary segment or Segment II) is immediately visible through the thin superior wall of the maxillary sinus (Fig. 4). It is a useful landmark to assist surgeon when performing procedure such as endoscopic transmaxillary approach to address the lesions within the anterolateral or retromaxillary spaces [27–29]. It is performed, either via a sublabial incision or a purely endonasal transmaxillary incision, and enables access to a wide range of anatomical targets within the infratemporal and pterygopalatine fossae such as lesions located laterally from the temporomandibular joint and the zygoma to the cavernous sinus and sella medially, as well as pathology located within the orbital floor [30, 31].

## Conclusions

The OF is constantly below the anterior skull base. It is a reliable and useful surgical landmark in endoscopic endonasal surgery. It is an important intraoperative reference point as a guide for safe posterior ethmoidectomy or sphenoidectomy dissection. By using the OF as a reference point, accidental injury to the skull base and other critical structures is avoided.

## Abbreviations

AEA: Anterior ethmoidal artery
CT PNS: Computed tomography of the paranasal sinuses
ESS: Endoscopic sinus surgery
IGS: Image-guided surgery
ION: Infraorbital nerve
OF: Orbital floor
PRISMA: Preferred reporting items for systematic reviews and meta-analyses
